# The information highways of a biotechnological workhorse – signal transduction in *Hypocrea jecorina*

**DOI:** 10.1186/1471-2164-9-430

**Published:** 2008-09-20

**Authors:** Monika Schmoll

**Affiliations:** 1 Research Area of Gene Technology and Applied Biochemistry, Institute for Chemical Engineering, Vienna University of Technology, Getreidemarkt 9/1665, A-1060 Wien, Austria

## Abstract

**Background:**

The ascomycete *Hypocrea jecorina *(anamorph *Trichoderma reesei*) is one of the most prolific producers of biomass-degrading enzymes and frequently termed an industrial workhorse. To compete for nutrients in its habitat despite its shortcoming in certain degradative enzymes, efficient perception and interpretation of environmental signals is indispensable. A better understanding of these signals as well as their transmission machinery can provide sources for improvement of biotechnological processes.

**Results:**

The genome of *H. jecorina *was analysed for the presence and composition of common signal transduction pathways including heterotrimeric G-protein cascades, cAMP signaling, mitogen activated protein kinases, two component phosphorelay systems, proteins involved in circadian rhythmicity and light response, calcium signaling and the superfamily of Ras small GTPases. The results of this survey are discussed in the context of current knowledge in order to assess putative functions as well as potential impact of alterations of the respective pathways.

**Conclusion:**

Important findings include an additional, bacterial type phospholipase C protein and an additional 6-4 photolyase. Moreover the presence of 4 RGS-(Regulator of G-protein Signaling) proteins and 3 GprK-type G-protein coupled receptors comprising an RGS-domain suggest a more complex posttranslational regulation of G-protein signaling than in other ascomycetes. Also the finding, that *H. jecorina*, unlike yeast possesses class I phosducins which are involved in phototransduction in mammals warrants further investigation. An alteration in the regulation of circadian rhythmicity may be deduced from the extension of both the class I and II of casein kinases, homologues of which are implicated in phosphorylation of FRQ in *Neurospora crassa*. On the other hand, a shortage in the number of the pathogenicity related PTH11-type G-protein coupled receptors (GPCRs) as well as a lack of microbial opsins was detected. Considering its efficient enzyme system for breakdown of cellulosic materials, it came as a surprise that *H. jecorina *does not possess a carbon sensing GPCR.

## Background

*Hypocrea jecorina *(anamorph *Trichoderma reesei*) was first isolated in the tropics during the Second World War and is found in soils, feeding on decaying wood and decomposing plant matter [1]. Since then strains of *H. jecorina *have been isolated from numerous habitats around the world. Its natural habitat indicates an evolution of *H. jecorina *towards recognition of substrates comprising cellulose and hemicellulose, which requires development of an adequate signal transduction machinery to optimize energy consumption against energy (substrate-) availability. This speciation led to the highly efficient cellulase and hemicellulase production of this fungus, which is presently also exploited by the biotechnological industry [2-4]. *H. jecorina *therefore has become a paradigm for the enzymatic breakdown of cellulose and hemicellulose, but is also known as a potent host for heterologous protein production due to its strong inducible promotors [5,6]. Despite the industrial utility and effectiveness of these carbohydrate-active enzymes of *H. jecorina*, a surprisingly small set of cellulases, hemicellulases and pectinases is available in its genome [7]. Also the number of enzymes comprising a carbonhydrate binding module in *H. jecorina *is the lowest among Sordariomycetes analysed so far. Nevertheless, this limited enzyme set obviously does not cause an evolutionary disadvantage in competition with other cellulose and hemicellulose-degrading fungi. Since in some cases in *H. jecorina *glycoside hydrolase genes are clustered near genes encoding proteins involved in secondary metabolite production, it is tempting to speculate that the success of *H. jecorina *might be connected to efficient control of expression of genes belonging to these groups [7].

Apart from studies on the properties of these enzymes and their regulation (for reviews see [5,6,8]), there has been a continuing interest in understanding the role of fungi in the carbon turn over in nature generally as well as of the physiology of the organism and its interrelationship with its environment. Accordingly, *H. jecorina *has recently also been used to study the significance of carbon catabolite derepression and cellulase gene expression for the antagonization of phytopathogenic fungi and suggested to be used as a model organism for related studies [9].

Crucial for the prosperity of any organism is the ability to survive under various conditions as well as to rapidly and appropriately react to a changing environment, which is mainly accomplished through an efficient signaling machinery [10,11]. The tight regulation of the numerous enzymes expressed by *H. jecorina *as well as its broad industrial applicability suggest a sophisticated signal transduction system, which provides the fungus with a tool kit for adjusting to such different environments as the soil of a tropic forest and a shake flask culture with a minimal medium. Nevertheless, while characteristics and regulation of the hydrolytic enzymes have been subject to numerous studies for decades now, analysis of the signal transduction machinery of *H. jecorina *is still in its start-up phase. To date, only one signal transduction protein has been analyzed on a molecular level: ENVOY, a PAS/LOV domain protein is crucial for light tolerance in *H. jecorina *and is involved in regulation of cellulase gene expression [12]. A recent study moreover indicates that this protein has a more widespread function than only regulation of processes related to light response in that it shows that ENVOY influences transcription of genes with various functions and does so not only in light but also during growth in darkness [13]. Besides that, only one earlier study dealt with a signaling event in *H. jecorina*: Mach and coworkers [14] showed that calmodulin-antagonists interfere with xylanase formation and secretion and thereby provided a first hint as to the significance of Ca^2+ ^signaling in this fungus.

However, the availability of the sequenced genome of *H. jecorina *[15] now opens new possibilities for investigating the potential of this fungus to perceive and interpret the signals from its environment. Gene modeling revealed 9129 gene models for *H. jecorina *[7], which is relatively close to the number of gene models in *N. crassa *[16], but is roughly 2500 fewer than the number of predicted genes in *Fusarium graminearum *[17]. Based on in depth analysis of the genome of *H. jecorina*, this study therefore intends to provide a basis for further investigation of signal transduction pathways by placing the *H. jecorina *orthologues of members of common signaling cascades described in other organisms in a genomic perspective. Characterized functions of those genes are shown together with the background of previous studies in *H. jecorina *and ascomycetes in general. Consequently a first map of the signaling landscape is laid out which shows both common highways as well as uncharted territories with islands reflecting the individuality of this organism.

## Results and Discussion

### Heterotrimeric G-protein signaling

Heterotrimeric G-proteins, comprising alpha-, beta- and gamma-subunits, are essential components of the signal transduction machinery in eukaryotic cells [18,19]. They transduce signals received by the heptahelical G-protein coupled receptors (GPCRs) from outside the cell and impact numerous regulatory pathways via their respective effectors, which in turn effect activity of secondary messengers [20,21]. The G-alpha subunit is tightly associated with the respective G-beta and -gamma subunit and binds GDP in its inactive state. Upon activation following binding of a ligand to its corresponding GPCR, GDP is exchanged for GTP and the G-alpha subunit dissociates from the G-beta-gamma heterodimer. Both parts are then free to activate their downstream targets [22]. With the increasing number of sequenced genomes of filamentous ascomycetes also a broad overview of the components of the heterotrimeric G-protein signaling pathway became available and several studies now provide a comprehensive summary on this topic (for example [23,24]). While heterotrimeric G-protein signaling has been studied extensively in several fungi including closely related *Trichoderma spp*. and has been implicated in such diverse processes as hyphal growth, conidiation, stress responses, chitinase formation, carbon source sensing, production of antifungal metabolites and mycoparasitic coiling [25-31] the functions of the *H. jecorina *homologues are not yet known.

**Figure 1:**
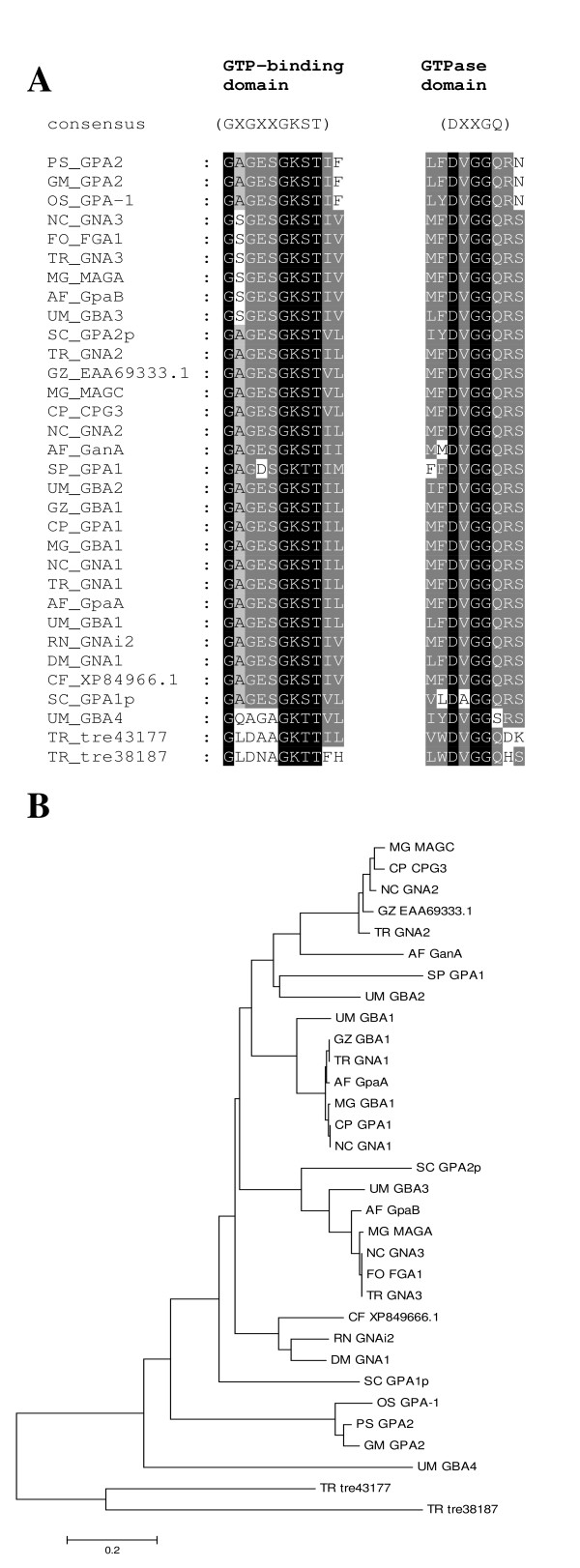
**Alignment and phylogenetic analysis of G-protein alpha subunits**. The *H. jecorina *genome comprises 3 G-alpha subunits belonging to the three subgroups common in other fungi. Accession numbers (corresponding database: GenBank) of the protein sequences used are those of the nearest neighbours of *Trichoderma reesei *(*Hypocrea jecorina*) (TR) in *Saccharomyces cerevisiae *(SC), *Schizosaccharomyces pombe *(SP), *Neurospora crassa *(NC), *Aspergillus fumigatus *(AF), *Gibberella zeae *(GZ), *Fusarium oxysporum *(FO), *Magnaporthe grisea *(MG), *Cryphonectria parasitica *(CP), *Rattus norvegicus *(RN), *Canis familiaris *(CF), *Drosophila melaongaster *(DM), *Oryza sativa *(OS), *Pisum sativum *(PS) and *Glycine max *(GM), are as listed in Table 1. Accession numbers (corresponding database: GenBank) of the G-protein alpha subunits of *Ustilago maydis *(UM) are: GPA1 P87032, GPA2 P87033, GPA3 P87034 and GPA4 P87035. (A) Alignment of GTP-binding domains and GTPase domains of G-protein alpha subunits including the two additional putative G-protein alpha subunits of *H. jecorina *(tre43177 and tre38187). While the GTPase domain of these two proteins matches the consensus (DXXGQ), the GTP binding domain is altered at the same position as in *U. maydis *Gpr4p (GX**G**XXGKS/T) [32]. (B) Phylogenetic analysis of G-alpha subunits using the Minimum evolution method and 500 Bootstrap replications as test of phylogeny.

Analysis of the genome revealed that *H. jecorina *has three G-alpha subunits, one G-beta subunit and one G-gamma subunit (Table 1), which corresponds well with the data of *Neurospora crassa *[25] and many other filamentous fungi [24]. However, considering the presence of 125 proteins comprising G-beta like WD-repeats in the genome of *H. jecorina*, of which 22 are not synthenic in *F. graminearum *[7], it cannot be excluded that further functional G-beta subunits exist, albeit as in other fungi, no evidence for interaction with G-alpha subunits or a function comparable to G-beta subunis of these proteins is available. While both G-alpha and G-beta subunits are well conserved in the fungal kingdom, the G-gamma subunit only shares low similarity to its nearest neighbours (Table 1). A phylogenetic analysis (Figure 1A) showed that the G-alpha subunits cluster well with the corresponding proteins of other fungi. The two closest homologues of the fourth G-alpha protein of *Ustilago maydis *[32], tre38187 and tre43177, which comprise GTP binding domains altered similarly to Gpa4 and unaltered GTPase domains (Figure 1B), were included in this analysis. However, these proteins did not cluster with Gpa4, but rather represent an outgroup in this tree. Consequently it is unlikely that tre38187 and tre43177 are functional G-alpha proteins, although experimental evidence remains to prove this hypothesis. Also a search for homologues of the fourth G-alpha subunit of *Aspergillus oryze*, GaoC [23] did not reveal a further G-alpha subunit in *H. jecorina*. Harashima and Heitmann [33] reported on G-protein beta mimics in *Saccharomyces cerevisiae *which contain 7 kelch repeats and bind to the G-alpha protein Gpa2p. These proteins (Gpb1p and Gpb2p) also assume a function in regulation of filamentous growth in *S. cerevisiae*. While initially considered G-beta and G-gamma mimics they are now rather considered effectors of Gpa2p [34]. In *H. jecorina*, several Kelch-repeat proteins have been found, but none of them contained 7 Kelch repeats or showed any similarity to Gpb1p or Gpb2p. Also for the described *S. cerevisiae *G-gamma mimic Gpg1p no homologue was found. Consistently, such G-beta or G-gamma mimics/G-alpha effectors have not been found in other filamentous fungi so far.

**Table 1:** Heterotrimeric G-protein signaling

	**Best hit overall**	***S. cerevisiae***	***S. pombe***	***Animal***	**Plant (Viridiplantae limited)**	***Neurospora***	***Aspergillus***	***Fusarium***	***Magnaporthe***	***Cryphonectria***
GNA1	*Hypocrea virens *G-protein alpha subunit, 0.0, 100% (AA018659.1)	Gpa2p, 2e-75, 43%, (NP_010937.1)	gpa1, 2e-71, 42%, (CAA21150.1)	*Rattus norvegicus *Gnai2, 4e-105, 55%, (NP_112297.1)	*Oryza sativa *GPA-1, 2e-52, 37% (AAV43839.1)	*Neurospora crassa *GNA-1, 0.0, 96%, (AAA02560.1)	*Aspergillus fumigatus *GpaA, 0.0, 94% (EAL90646.1)	*Gibberella zeae *PH-1, GBA1, 0.0, 98% (EAA75079.1)	*Magnaporthe grisea MAGB*, 0.0, 97%, (AAB65426.1)	*Cryphonectria parasitica *GPA1, 0.0, 98%, (Q00580)
GNA2	*Hypocrea virens *G-protein alpha subunit, 0.0, 94% (AAN65182.1)	Gpa2p, 3e-66, 41%, (NP_010937.1)	gpa1, 7e-82, 47%, (CAA21150.1)	*Canis familiaris *Guanine nucleotide-binding protein G(t), alpha-3 subunit, 8e-89, 48%, (XP849666.1)	*Pisum sativum *GPA2, 3e-52, 35%, (AAM97353.1)	*Neurospora crassa *GNA-2, 4e-163, 78% (CAD71253.1)	*Aspergillus fumigatus *GanA, 3e-105, 53% (EAL92343.1)	*Gibberella zeae *PH-1, hypothetical protein FG09988.1, 1e-169, 79% (EAA69333.1)	*Magnaporthe grisea *MAGC, 9e-162, 77%, (AAB65427.1)	*Cryphonectria parasitica *CPG-3, 6e-163, 77%, (AAM14395)
GNA3	*Trichoderma atroviride *Tga3, 0.0, 96% (AAM69919.1)	Gpa2p, 8e-105, 53%, (NP_010937.1)	gpa2, 2e-83, 45%, (Q04665)	*Drosophila melanogaster *GNA1, 4e-88, 48%, (P20353)	*Glycine max *GPA-2, 5e-54, 33% (P93163)	*Neurospora crassa *GNA-3, 0.0, 91% (EAA76506.1)	*Aspergillus fumigatus *GpaB, 1e-154, 75% (EAL90625.1)	*Fusarium oxysporum *fga1, 0.0, 90% (BAD44729.1)	*Magnaporthe grisea *MAGA, 0.0, 87%, (AAB65425.1)	*Cryphonectria parasitica *GPA1, 1e-93, 49%, (Q00580)
GNB1	*Trichoderma atroviride *TGB1, 0.0, 98%, (AA037685.1)	Ste4, G-protein beta subunit, 1e-72, 37% (P18851)	gpb1, 3e-73, 44%, (Q10282)	*Caenorhabditis elegans *gpb-1, 2e-137, 69% (P17343)	*Oryza sativa *GBB, 1e-97, 48% (Q40687)	*Neurospora crassa *GNB-1, 0.0, 91% (AAM53552.1)	*Aspergillus nidulans *GBB, 5e-174, 84% (XP_657685.1)	*Fusarium oxysporum *fgb1, 0.0, 98%, (AA091808.1)	*Magnaporthe grisea *Mgb1, 0.0, 95% (BAC01165.1)	*Cryphonectria parasitica *GB-1, 0.0, 94% (O14435)
GNG1	*Gibberella zeae *PH-1, conserved hypothetical protein, 2e-35, 95% (EAA77833.1)	None	None	None	None	*Neurospora crassa *GBG1, 5e-25, 81% (Q7RWTO)	*Aspergillus nidulans *hypothetical protein AN2742.2, 2e-17, 67% (XP_660346.1)	*Gibberella zeae *PH-1, conserved hypothetical protein, 2e-35, 95% (EAA77833.1)	*Magnaporthe grisea *hypothetical protein MG10193.4, 6e-32, 84% (XP_365973.1)	None

Nearest neighbours of the respective organisms are listed along with E-value as determined by NCBI blastp search, percent identities within fragment and GenBank accession number.

#### G-protein coupled receptors

The signal to be transmitted through a heterotrimeric G-protein signaling cascade is received by the plasma-membrane localized G-protein coupled receptors. Screening the *H. jecorina *genome for heptahelical transmembrane G-protein coupled receptors revealed a total of 34 such proteins. They represent members of all but two classes of GPCRs defined in filamentous fungi [24] (Table 2). Besides the two pheromone receptors (tre18617 and tre19688), two members of the class of Stm1-like putative nitrogen sensors and one orthologue of the rat growth hormone releasing factor homologue MG00532 of *Magnaporthe grisea *were detected. However, in contrast to other ascomycetes, no members of the group of microbial opsins or an opsin-related protein similar to *N. crassa *NOP-1 or ORP-1 is present. These proteins function as light responsive ion pumps or sensory receptors [25]. Analysis of *N. crassa *NOP-1 revealed a function in modulation of carotenogenesis and regulation of conidiation specific genes and represents a putative green light photoreceptor, which is regulated by WC-2 [35-37]. The significance of this shortcoming in *H. jecorina *is difficult to assess, since despite a lack of banding without light pulses, conidiation still responds to light and this fungus is not reported to produce carotenoids. Also a GPCR similar to the glucose sensors found in *S. cerevisiae, S. pombe *and the carbon sensor GPR-4 of *N. crassa *[24,25,38,39] is not present in the genome of *H. jecorina*. The genomic locus of the *H. jecorina *homologue of *N. crassa *GPR-4 (NCU06312.3) obviously has been lost, because the genes flanking the locus of this receptor in *N. crassa *(NCU06311.3 and NCU06314.2) are synthenic in *H. jecorina*, *N. crassa *and *F. solani *[40], while GPR-4 and NCU06313.3 have no homologues in *H. jecorina*. Instead, it seems that an insertion has occurred resulting in a non synthenic region comprising putative genes with low similarity to genes of filamentous fungi. On the other hand, the groups of cAMP-receptor like proteins (5 members) as well as the group of GPCRs related to the *H. sapiens *mPR-like protein (5 members) and the class of *Aspergillus *GprK-like receptors [23], which comprise an RGS domain (3 members), are expanded in comparison with other filamentous fungi [24]. The large family of PTH11-receptors (21 members in *N. crassa *and considerably more in the plant pathogens *Magnaporthe grisea *(60) and *Fusarium graminearum *(105)) is present with numbers in the range of the saprophytic *N. crassa *and thus clearly underrepresented in *H. jecorina *with only 16 members as compared to pathogenic fungi. This is the lowest number of these proteins present in the filamentous fungi analyzed to date [24]. The shortage of these GPCRs originally identified as pathogenicity-related in *Magnaporthe grisea *[41,42] could be indicative of a saprophytic/non pathogenic life style considering the also lower number of these receptors in *N. crassa*.

**Table 2:** G-protein coupled receptors of *H. jecorina*

**function**	**type**	**ID**
**Pheromone receptors**	Ste2	tre18617
	
		Ste3	tre19688

**cAMP-receptor like proteins**		tre29339
		tre36247
		tre59778
		tre72004
		tre72627

**carbon sensors**		none

**putative nitrogen sensors**	Stm1-like	tre80125
		tre4508

**microbial opsins**		none

**related to *H. sapiens *mPR- like proteins**	mPR-like	tre56426
		tre68212
		tre70139
		tre82246
		tre119819

**related to rat growth hormone releasing factor**	*M. grisea *MG00532-like	tre456

**GPCR comprising RGS-domain**	*Aspergillus *GprK-like	tre37525
		tre63981
		tre81383

**implicated in pathogenesis in *Magnaporthe grisea***	PTH11-type	tre5647
		tre27983
		tre27992
		tre41260
		tre45573
		tre55561
		tre57101
		tre62462
		tre69500
		tre76763
		tre103694
		tre107042
		tre110339
		tre111861
		tre121990
		tre124113

Classification was applied according to [24]

#### Regulation of G-protein signaling

**Figure 2:**
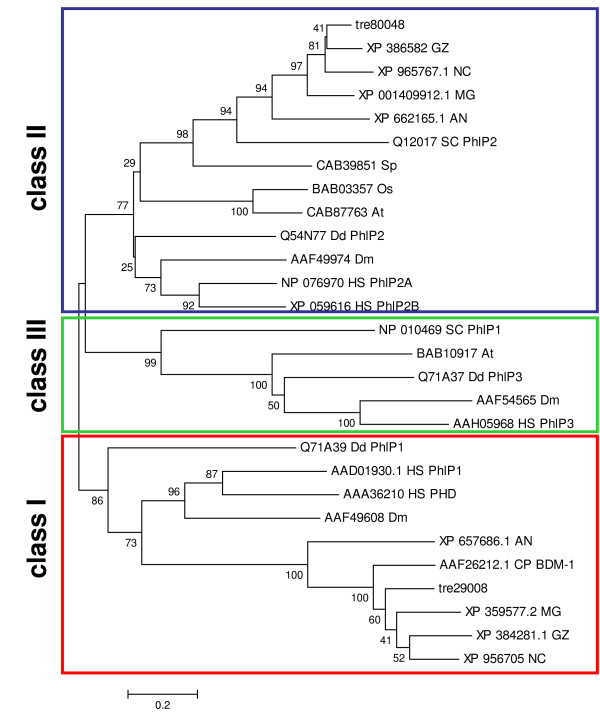
**Phylogenetic analysis of phoducin-like proteins**. In contrast to *S. cerevisiae*, *H. jecorina *and other filamentous ascomycetes possess class I phosducin-like proteins, but no class III phosducin-like proteins. GenBank accession numbers of the protein sequences used are those of the nearest neighbours of *Trichoderma reesei *(*Hypocrea jecorina*) (treXXXXX) in *Saccharomyces cerevisiae *(SC), *Schizosaccharomyces pombe *(SP), *Neurospora crassa *(NC), *Aspergillus nidulans *(AN), *Gibberella zeae *(GZ), *Magnaporthe grisea *(MG), *Cryphonectria parasitica *(CP), *Dictyostelium discoideum *(Dd), *Drosophila melaongaster *(DM), *Oryza sativa *(OS), *Homo sapiens *(HS) and *Arabidopsis thaliana *(At). The analysis was performed using the Minimum Evolution method and 500 Bootstrap replications as test of phylogeny.

**Figure 3:**
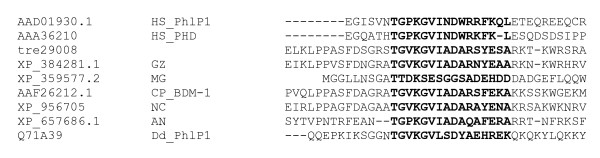
**Alignment of regions similar to the G-beta-gamma binding conserved helix 1 of *Homo sapiens *PhlP1 and Pdc in filamentous ascomycetes**. The conserved sequence is given in bold for *Trichoderma reesei *(*Hypocrea jecorina*) (treXXXXX), *Neurospora crassa *(NC), *Aspergillus nidulans *(AN), *Gibberella zeae *(GZ), *Magnaporthe grisea *(MG), *Cryphonectria parasitica *(CP), *Dictyostelium discoideum *(Dd), and *Homo sapiens *(HS).

Regulation of the intensity of a G-protein mediated signal is mainly accomplished by the action of phosducins or regulators of G-protein signaling (RGS-proteins) [43-45]. Phosducins modulate the activity and availability of the G-beta-gamma complex and are reported to be positive regulators of G-beta function. However, recent data overturned the initial hypothesis [46,47] that phosducin would block G protein signaling by disrupting the interaction between G-protein alpha subunits and the G-betagamma dimer. The current understanding of phosducin-function rather suggests a regulatory influence as an essential cochaperone of G-beta-gamma folding [48-50]. In filamentous fungi two studies in *Cryphonectria parasitica *[44] and *Aspergillus nidulans *[45] revealed that deletion of the respective phosducin results in a phenotype similar to deletion mutants in the respective G-beta subunit, an effect also seen with *Dictyostelium discoideum *[51]. In order to assess the position of the two *H. jecorina *phosducins (tre80048 and tre29008) within the classification as defined by Blaauw et al., [51], a phylogenetic analysis with a subset of the proteins used in this study together with the phosducin-homologues of several ascomycetous fungi and those of *H. jecorina *was performed (Figure 2). This analysis revealed that filamentous ascomycetes possess members of the class II phosducins, but – in contrast to *S. cerevisiae *– they also have class I phosducins, whereas only in *A. nidulans *members of class III of phosducin-like proteins as in *S. cerevisiae *were found (Table 3). These class I phosducins – with the exception of the *M. grisea *protein – also comprise an N-terminal region with high similarity to the 11 aa sequence conserved between the two *Homo sapiens *subgroup I phosducins Pdc and PhlP1 [52], which is a major interaction site of the transducin G-betagamma dimer [53] (Figure 3). Consequently, it remains to be shown whether the function of these proteins is executed as cochaperones as could be assumed if they are homologues of PhlP1 or rather resembles the function of Pdc, i. e. to ensure efficient translocation of the G-beta-gamma dimer during light and dark adaptation [50]. Since light response or -adaptation has not been reported in *S. cerevisiae *and homologues of subclass I have not been detected in this ascomycete, the elaborate studies on yeast phosducins [54] cannot contribute to an elucidation of the underlying mechanism in other, filamentous ascomycetes.

**Table 3:** Classification of phosducin proteins in ascomycetes as deduced from phylogenetic analysis

**Organism**	**Class I**	**Class II**	**Class III**
*Hypocrea jecorina*	tre29008	tre80048	
*Gibberella zeae*	XP_384281.1	XP_386582	
*Magnaporthe grisea*	XP_357577.2	XP_001409912.1	
*Neurospora crassa*	XP_956705	XP_965767.1	
*Aspergillus nidulans*	XP_657686.1	XP_662165.1	EAA60135
*Saccharomyces cerevisiae*		Q12017	NP_010469

Regulators of G-protein signaling have an enhancing effect on the intrinsic GTPase activity of G-alpha subunits and thus can cause termination of the mediated signal [55]. Comparably to *A. nidulans *[56], *H. jecorina *possesses orthologues of all four RGS-proteins of *A. nidulans *(tre38047, tre78314, tre65607 and RGS1 corresponding to RgsA, RgsB, RgsC and FlbA, respectively). Interestingly, *H. jecorina *also possesses 3 homologues of the *A. nidulans *RGS-domain containing G-protein coupled receptor GprK (tre37525, tre63981 and tre81383), which is the highest number of these proteins in all fungi screened to date [24]. Consequently, it appears that *H. jecorina *applies tight control on the signals to be transmitted via the heterotrimeric G-protein signaling cascade. That could indicate that *H. jecorina *executes a thorough determination of the significance of the respective signal for its downstream targets under certain environmental conditions.

Adjustment of the duration of a GPCR-mediated signal is executed by β-arrestins, which have predominantly been studied in higher organisms [57,58]. They contribute to the desensitization of the GPCR after phosphorylation by G-protein coupled receptor kinases (GRKs) and thus promote termination of the G-alpha signal. Two candidate genes encoding potential β-arrestins were detected (tre14071 and tre39560), which have homologues in *Gibberella zeae, M. grisea*, *N. crassa *and *A. nidulans*. Among them are the *A. nidulans *pH-response regulator protein PalF (E-value 2E-103; [59]) and the *A. nidulans *carbon catabolite repressor protein CreD (E-value 2E-72; [60]). However, despite several proteins related to mammalian G-protein coupled receptor kinases, no protein of *H. jecorina *bearing a GPCR kinase domain (IPR000239) was found. Therefore the mentioned phosphorylation step might be executed by a different kinase or alternatively the *H. jecorina *GRKs share only low homology with GRKs characterized so far.

### cAMP signaling

Cyclic AMP was found to activate a variety of glucose-repressed functions, including catabolism of carbon sources but also to control numerous often apparently unrelated functions in animal cells. Thus cAMP was initially proposed as a primitive signal for carbon starvation, although also its effects on development and other specific responses have been subject to extensive analyses since. In fungi, its effects reach from the control of utilization of endogenous and exogenous carbon sources and conidiation in *N. crassa*, dimorphism and sexual development in several fungi and phototropism in *Phycomyces *[10,61]. Addition of cAMP to cultures of *Trichoderma atroviride *induces mycoparasitism-related coiling around simulated host-hyphae even in the absence of lectins [28]. Crosstalk of the cAMP signaling pathway with the light perception machinery has been reported in *T. atroviride *[62,63]. In *H. jecorina *addition of cAMP can double the efficacy of sophorose induction of endoglucanase formation [64], but this effect decreases if the concentration exceeds the optimum. In yeast, the addition of a rapidly fermented sugar to derepressed cells initiates a rapid, transient spike in the cAMP level [65] and intracellular acidification stimulates cAMP synthesis [66], although the respective physiological role has remained enigmatic. The studies of Farkas and coworkers [67], Sestak and Farkas [64] and Montenecourt [68] on the correlation of intracellular cAMP-levels and cellulase formation during growth on different carbon sources did not yield consistent results. Nevertheless, they suggest that cAMP might be an important signal for regulation of cellulase formation, but not the only one. Analysis of intracellular cAMP-levels revealed a significant increase in response to a light pulse [69], which is in concordance with the finding of a light activated adenylyl cyclase in *Trichoderma viride *[70]. This intracellular concentration of cAMP is determined by the balance between the activities of adenylyl cyclase and 3'5' cyclic AMP phosphodiesterase. In yeast, Ras small GTPases have been implicated in regulation of the cAMP pathway and suggested to regulate adenylate cyclase [66]. However the precise mechanism of this influence has not been established yet. The importance of cAMP signaling for *H. jecorina *is highlighted by the finding of 5 putative cAMP-GPCRs. However, the reception of cAMP as an environmental signal by a GPCR in filamentous fungi remains to be proven.

The genome of *H. jecorina *comprises one adenylyl cyclase (ACY1), one adenylyl cyclase associated protein (tre22793), one low affinity cyclic nucleotide phosphodiesterase (tre35876) and one high affinity cyclic nucleotide phosphodiesterase (tre32709). Similarly to *N. crassa*, *H. jecorina *possesses 2 protein kinase A catalytic subunits (PKAC1 and PKAC2) and one regulatory subunit (PKAR1). The hypothesis that cAMP or a related molecule could act as an environmental signal and initiate a signaling cascade by binding to a GPCR as suggested for *N. crassa *[25] is corroborated by the findings for *H. jecorina*, albeit such a function has not yet been shown for any filamentous fungus.

### Mitogen activated protein kinases (MAP kinases)

Mitogen activated protein kinase (MAP kinase) pathways represent one of the most prominent signal transduction systems. The respective signaling cascades comprise three serine/threonine protein kinases that act in series: MAP kinase kinase kinase (MAPKKK), MAP kinase kinase (MAPKK) and MAP kinase (MAPK) [71,72]. The signaling output of these cascades often targets transcription factors and is reported to be interconnected with cAMP-signaling [10,73] and heterotrimeric G-protein signaling [74,75]. MAP-kinases are essential for appressorium formation and virulence in *M. grisea *[76,77] and for fungal pathogenicity [78,79]. On the other hand, MAP-kinases are also important for plant systemic resistance, biocontrol of pathogenic fungi and mycoparasitism as shown for several *Trichoderma *spp. [80-83]. In *N. crassa *functions range from sexual development and hyphal fusion [84,85], osmosensitivity and resistance to fungicides [86-88] to circadian rhythmicity via the HOG1-orthologue OS-2 [89]. The most extensively studied example of the MAP kinase signaling system are the pathways of *S. cerevisiae *[71,72], the major functions of which are in osmosensing, filamentation, cell integrity, spore wall assembly and pheromone signaling.

**Figure 4:**
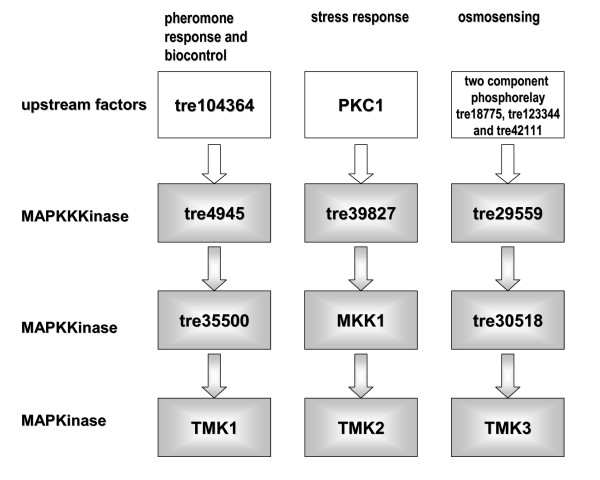
**Model for proposed MAP kinase cascades**. Pathways are given as deduced from reported functions and interactions of the respective nearest neighbours in *S. cerevisiae *and other filamentous ascomycetes. Therefore this model should be considered only a suggestion for further research needed to confirm these pathways.

Based on their homologues in *S. cerevisiae *and the pathways determined in this fungus [72] as well as according to the interaction partners of the respective kinases as determined using the Database of interacting proteins (DIP services [90]) a model for the putative MAP kinase cascades in *H. jecorina *was assembled (Figure 4). In *H. jecorina*, nine members of MAP-kinase cascades were identified, none of which has been characterized yet. The functions suggested for these pathways were assigned based on characterized homologues of the members of the cascades. Interestingly, in contrast to *S. cerevisiae*, which comprises five MAP kinase cascades, *H. jecorina *– similar to other filamentous ascomycetes – apparently only has three, for which functions in (i) pheromone response and biocontrol, (ii) stress response and protein degradation and (iii) osmosensing and potentially carbon source sensing are predicted. Homologues of the MAP kinases Kss1p, involved in filamentation and invasion as well as Smk1p, involved in spore cell wall assembly were not detected. The MAP kinase cascade assigned to pheromone response and biocontrol comprises the MAPK TMK1, the MAPKK tre35500 and the MAPKKK tre39827. TMK1 represents the *H. jecorina *homologue of Fus3p (E-value 1e-122) and shares high similarity with *Hypocrea virens *TMKA (AAN34610) and *T. atroviride *TMK1 (AAM69918), which are involved in biocontrol [80,82] as well as with the *M. grisea *pathogenicity MAP kinase Pmk1 (AAC49521; [76]). The MAPKK tre35500 shares highest similarity to *S. cerevisiae *Ste7p (E-value 5e-65) and is related to *Glomerella cingularia *EMK1 (AAD55386; E-value 2e-178), which is required for appressorium formation in this fungus [91]. tre4945 was assigned to this pathway because of its similarity to Ste11p (E-value 1e-90). However, due to the interaction of Ste11p also with Hog1p and Pbs2p a participation of this factor in osmosensing should be considered. The second MAP kinase cascade consists of TMK2, MKK1 and tre39827. TMK2 is related to *S. cerevisiae *Slt2p (E-value 1e-122), which is the MAP kinase of the stress response pathway, MKK1 shares high similarity to Mkk1p and tre39827 is the *H. jecorina *orthologue of Bck1p, which is a target for phosphorylation by protein kinase C (*Hypocrea jecorina *orthologue: PKC1). An orthologue corresponding to the protein redundant to Mkk1p, Mkk2p, was not detected in *H. jecorina*. The interactions of Slt2p with the 26S proteasome regulatory protein Rpt3p and of Mkk1p with the 26S proteasome regulatory subunit Rpn1p suggest a role for this pathway also in regulation of protein degradation. In this respect it should be considered that MAP kinase pathways themselves can be subject to regulation by ubiquitin mediated degradation [92]. A potential autoregulatory cycle in MAP kinase signaling could thus be worth exploring. The pathway assigned to osmosensing comprises TMK3 (E-value of 1e-175 to Hog1p), tre30518 (E-value of 1e-98 to Pbs2p) and tre29559 (E-value of 1e-140 to Ssk2p). The interactions of the TMK3 orthologue with Sko1, a hydrolase regulating transcription factor and of the tre29559 orthologue with the CCAAT-binding factor Hap2p, the *H. jecorina *orthologue of which binds to the cellulase activating element CAE within the *cbh2*-promotor [93], suggest a role of this pathway also in carbon source signaling. Moreover, the *tmk3*-transcript was shown to be responsive to light and regulated by ENVOY [13], which – considering the involvement of the *N. crassa *homologue OS-2 in circadian rhythmicity [89] – warrants a more detailed analysis of its function in *H. jecorina*.

### p21 activated kinases (PAKs)

Members of this protein kinase family are able to modulate MAP kinase pathways and can be activated by binding to GTP-bound Rho-like GTPases of the Cdc42/Rac family [94,95]. PAKs characteristically comprise a serine/threonine protein kinase domain at the C-terminus and a CRIB (Cdc42/Rac interactive binding)-domain at the N-terminus. The CRIB domain can bind the serine/threonine kinase domain and thereby inhibit its activity. This inhibition can be abolished by binding of a GTP bound Rho-like GTPase to the CRIB domain. The best characterized member of this family of kinases, *S. cerevisiae *Ste20p, is often referred to as MAP4K because of its regulatory impact on the Fus3 MAP kinase pathway. Ste20p is further involved in pheromone response, response to osmotic stress, filamentation and polarized growth and links the pheromone response G-protein beta gamma subunits to downstream signaling components [96,97]. Cla4p, the second PAK of *S. cerevisiae *is involved in budding and cytokinesis [98]. The *H. jecorina *genome comprises homologues of the *S. cerevisiae *PAKs Ste20p (tre104364) and Cla4p (tre28344). Both tre104364 and tre28344 also contain the characteristic CRIB-domain at the N-terminus.

### Germinal center kinases (GCKs)

Germinal center kinases represent the second group of Ste20-like protein kinases [99]. These kinases are related to the human germinal center kinase (GCK) and are implicated in the regulation of stress activated MAPK signaling pathways. In contrast to the p21 activated kinases, their serine/threonine kinase domain is present at the N-terminus of the protein and is followed by a poorly conserved sequence lacking CRIB or PH domains. These C-terminal sequences may contribute to the autoinhibition of the kinase activity, which can be counteracted by binding of other components. *H. jecorina *contains one candidate germinal center kinase related to *S. cerevisiae *Sps1p (tre45283) and three candidates related to *S. cerevisiae *Kic1p (tre30044, tre61703, tre43532). Due to the low conservation within this family, assigning functions to these proteins and confirmation that they indeed are germinal center kinases will only be possible after functional characterization.

### Two component phosphorelay systems

**Figure 5:**
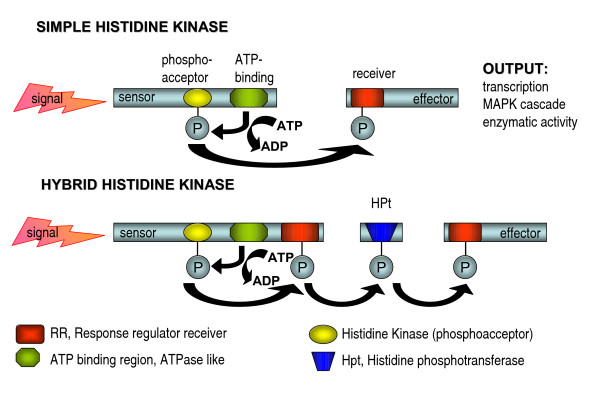
**Schematic representation of two component phosphorelay systems **(adapted from [180]). In response to an environmental signal the two component phosphorelay signaling cascade is initiated by ATP-dependent autophosphorylation of the histidine kinase (HK) at a conserved histidine residue. Then this phosphate is transferred to a conserved aspartic acid within a response regulator (RR) domain, which ultimately causes a change in transcription of the respective target gene or regulation of a mitogen-activated protein kinase pathway [101].

Two component histidine kinase phosphorelay systems represent conserved signaling pathways employed by both prokaryotes and eukaryotes to adapt to changes in their environment. Components of this system have been characterized from bacteria, slime moulds, plants and fungi, but they were not found in animals. Their functions range from differentiation, chemotaxis, secondary metabolite production and virulence to adaptation to osmotic stress [100]. However, since there are classes of histidine kinases for which a physiological function remains to be identified, this list may still be incomplete. Signaling via two component phosphorelay systems in response to an environmental signal is initiated by ATP-dependent autophosphorylation of the histidine kinase (HK) at a conserved histidine residue. Then this phosphate is transferred to a conserved aspartic acid within a response regulator (RR) domain, which ultimately causes a change in transcription of the respective target gene or regulation of a mitogen-activated protein kinase pathway [101]. Two different types of two component signaling have been described: With the simple histidine kinases the sensor histidine kinase and the regulator receiver are separate proteins, whereas hybrid histidine kinases contain both HK and RR domains on the same protein and generally require additional rounds of phosphorelay through a histidine phosphotransferase (HPt) and another RR protein (Figure 5). In fungi to date only hybrid histidine kinases have been identified and in general they are predominant in eukaryotes, while simple HKs are predominant in prokaryotes.

Although components of phosphorelay systems have been analysed from several organisms, the most comprehensive survey on the mechanism is available from *S. cerevisiae*: The sole yeast histidine kinase Sln1p is part of the two component regulatory system Sln1p/Ssk1p and becomes activated by changes in osmolarity of the extracellular environment. Sln1p, the signal receiver becomes autophosphorylated at an internal histidine residue. This phosphate is then intramolecularly transferred to an aspartic acid residue and finally to a histidine in the histidine phosphotransferase Ypd1p. Thereafter phosphotransfer occurs to an aspartic residue in the response regulator Ssk1p. This phosphorelay system targets the Ssk2p-Pbs2-Hog1 MAP kinase cascade, which also has an equivalent in *H. jecorina*. As long as Sln1p is incative, the unphosphorylated Ssk1p can activate the MAP kinase kinase kinase Ssk2p, which can stimulate the Pbs2p-Hog1p cascade in order to induce glycerol synthesis in the cell. Upon decreased osmolarity, the activation of this MAP kinase cascade is repressed due to activation of Sln1p, which phosphorylates Ssk1p [102].

In *N. crassa *the response regulator protein RRG-1 is involved in control of vegetative cell integrity, hyperosmotic sensitivity, fungicide resistance and protoperithecial development through regulation of the osmosensitivity MAP kinase pathway [87]. RRG-2, the second response regulator protein of *N. crassa*, is involved in oxidative stress response and the histidine phosphotransferase HPT-1 is assumed to negatively regulate a downstream MAP kinase pathway [103]. As for the hybrid histidine kinases, members of class III are among the best studied in filamentous fungi and are implicated mainly in osmosensing, their target being the respective MAP kinase cascade [104,105]. In *A. nidulans*, a recent study showed that at least some of its histidine kinases are both spatially and temporally differentially expressed during the cell cycle [106]. The response regulators of this fungus are involved in stress signaling and asexual sporulation [107].

**Figure 6:**
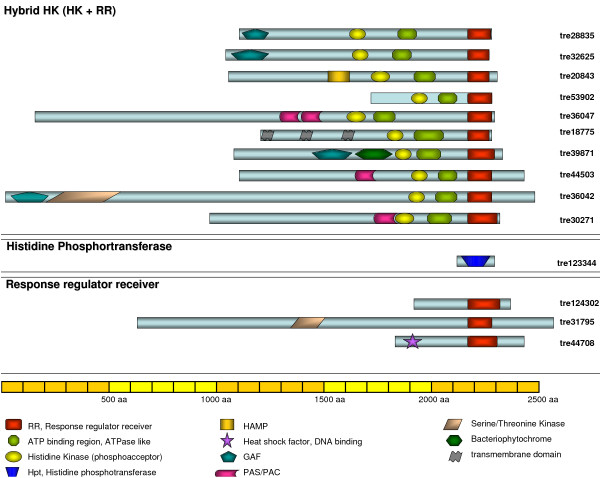
**Domain structure of two component phosphorelay histidine kinases and their putative interactors in *H. jecorina***. Models of the predicted proteins as annotated in the *Trichoderma reesei *Genome database v2.0 are drawn to scale. Position and significance of the respective domains was determined by InterPro search and NCBI CDD search.

Genome analysis of *H. jecorina *revealed the presence of ten putative histidine kinases, all of them hybrid HKs (Figure 6) as characterized by the presence of a response regulator receiver domain (IPR001789), a histidine kinase domain (IPR005467) and an ATP binding region (IPR003594). Further domains present in the HKs of *H. jecorina *were GAF-domains (IPR003018), which are often found in phytochromes, a HAMP-region (IPR003660) charakteristic for signaling HKs, PAS domains (IPR000014) implicated in sensing light, oxygen or voltage and in one case a bacteriophytochrome domain (IPR009219). These various domains allow for the classification of histidine kinases as described in the following.

According to the classification of Catlett et al., [108] the ten histidine kinases of *H. jecorina *belong to nine different classes (Table 4) and only members of classes II and VII, which comprise specialized HKs of *C. heterostrophus, B. fuckeliana *and *G. moniliformis*, but no orthologues of other fungi, are missing. As the functions of these genes are not known, the significance of their presence or absence remains obscure. Among the classes covered by members from *H. jecorina *are also those which contain closely related sequences from each species analyzed and are thus likely to represent the core HKs of fungi (groups III, V, VI, VIII, IX and X). The distribution of HKs among the groups varies between *N. crassa *and *H. jecorina*. While the *Neurospora *genome comprises two phytochromes (class VIII) and two members of group XI, *H. jecorina *only has one phytochrome (tre39871) and one member of group IX (tre30271). Interestingly, both groups are characterized by the presence of PAS-domains and hence this might reflect a more elaborate application of ligand- and presumably FAD-mediated signaling in *N. crassa *than in *H. jecorina*. Nevertheless, the finding of a phytochrome indicates that *H. jecorina *is able to sense and respond to the presence of red and far-red light [109]. However, the precise function of the phytochromes in light response of fungi remains to be determined, since deletion of the *N. crassa *phytochrome genes *phy-1 *and *phy-2 *did not compromise any known photoresponse [110]. Also in *C. heterostrophus *phytochrome deletion did not yield a discernible phenotype [108]. On the other hand, studies in *A. nidulans *revealed that the phytochrome FphA represses sexual development and mycotoxin formation in red light and that it is part of a complex comprising LreA and LreB, the homologues of *N. crassa *WC-1 and WC-2. Moreover FphA interacts with the light dependent regulator protein VeA [111-113]. *H. jecorina *possesses two class I HKs (tre28835 and tre32625) in contrast to one in *N. crassa*. Due to the function of the *S. cerevisiae *class I HK Ssk1p in osmoregulation, a similar function can be assumed in *H. jecorina*.

**Table 4:** Two component phosphorelay systems

**Protein class**	**protein ID**	**group**	**Best hit overall**	**Best hit non Trichoderma**	***S. cerevisiae***	***S. pombe***	**Plant (Viridiplantae limited)**	***Neurospora***	***Aspergillus***	***Fusarium***	***Magnaporthe***
Histidine kinase	tre28835	**I**	*Gibberella moniliformis *putative histidine kinase M27Mp, 0.0, 41% (AAR30136.1) 1190 aa		None	hypothetical protein SPAC1834.08, 6e-13, 33% (CAB75776.1) 1639 aa	*Arabidopsis thaliana *histidine kinase-like protein, 5e-13, 34% (CAC08246.1) 950 aa	*Neurospora crassa *putative two component histidine kinase NCU09520, 1e-99, 30% (DAA02217.1) 1529 aa	*Aspergillus fumigatus *sensor histidine kinase/response regulator, 2e-135, 31% (EAL84386.1) 1170 aa	*Gibberella moniliformis *putative histidine kinase M27Mp, 0.0, 41% (AAR30136.1) 1190 aa	*Magnaporthe grisea *hypothetical protein MG02897.4, 2e-13, 31% (XP_366821.1) 833 aa
Histidine kinase	tre32625	**I**	*Gibberella moniliformis *putative histidine kinase M27Mp, 0.0, 38% (AAR30136.1) 1190 aa	*Gibberella moniliformis *putative histidine kinase M27Mp, 0.0, 38% (AAR30136.1) 1190 aa	cytoplasmic response regulator Ssk1p, 1e-13, 32% (NP_013106.1) 712 aa	hypothetical protein SPAC1834.08, 2e-15, 36% (NP_594687.1) 1639 aa	*Arabidopsis thaliana *histidine kinase-like protein, 5e-12, 32% (CAC08246.1) 950 aa	*Neurospora crassa *hypothetical protein NCU09520.1, 5e-89, 29% (DAA02217.1) 1529 aa	*Aspergillus nidulans *histidine kinase G7, 1e-117, 29% (AAQ19475.1) 1282 aa	*Gibberella moniliformis *putative histidine kinase M27Mp, 0.0, 38% (AAR30136.1) 1190 aa	*Magnaporthe grisea *hypothetical protein MG02897.4, 3e-12, 31% (XP_366821.1) 833 aa
Histidine kinase	tre20843	**III**	*Fusarium solani *histidine kinase FIK, 0.0, 89% (AAD09491.1) 1283 aa	*Fusarium solani *histidine kinase FIK, 0.0, 89% (AAD09491.1) 1283 aa	nuclear response regulator and transcription factor Skn7p, 1e-14, 32% (NP_012076.1) 622 aa	None	None	*Neurospora crassa *Os-1p, 0.0, 80% (AAB01979.1) 1298 aa, corresponding to NCU02815.1	*Aspergillus fumigatus *two-component osmosensing histidine kinase Bos1, 0.0, 66% (EAL87451.1) 1377 aa	*Fusarium solani *histidine kinase FIK, 0.0, 89% (AAD09491.1) 1283 aa	*Magnaporthe grisea *histidine kinase, 0.0, 79% (BAB40947.1) 1307 aa
Histidine kinase	tre53902	**IV**	*Neurospora crassa *related to histidine kinase tcsA protein, 0.0, 62% (CAD71062.1) 922 aa	*Neurospora crassa *related to histidine kinase tcsA protein, 0.0, 62% (CAD71062.1) 922 aa	None	None	*Oryza sativa *putative sensor kinase PK4, 1e-59, 36% (AAP07255.1) 653 aa	*Neurospora crassa *related to histidine kinase tcsA protein, 0.0, 62% (CAD71062.1) 922 aa	*Aspergillus parasiticus *histidine kinase 7e-163, 52% (AAR92365.1) 708 aa	*Gibberella zeae *hypothetical protein FG05866.1, 1e-61, 35% (EAA75641.1) 2544 aa	*Magnaporthe grisea *hypothetical protein MG01342.4, 6e-55, 33% (EAA55691.1) 1388 aa
Histidine kinase	tre36047	**V**	*Gibberella moniliformis *putative histidine kinase HHK2p, 0.0, 62% (AAR30137.1) 2072 aa	*Gibberella moniliformis *putative histidine kinase HHK2p, 0.0, 62% (AAR30137.1) 2072 aa	None	hypothetical protein SPAC1834.08, 1e-76, 49% (CAB75776.1) 1639 aa	*Oryza sativa *putative sensor kinase PK4, 4e-41 (AAQ07255.1)	*Neurospora crassa *related to two component histidine kinase chk-1, 0.0, 57% (CAD70476.1) 2177 aa	*Aspergillus nidulans *hypothetical protein AN3101.2, 0.0, 35% (XP_660705.1) 1879 aa	*Gibberella moniliformis *putative histidine kinase HHK2p, 0.0, 62% (AAR30137.1) 2072 aa	*Magnaporthe grisea *histidine kinase, 4e-47, 44% (XP_363718.1) 1307 aa
Histidine kinase	tre18775	**VI**	*Neurospora crassa *related to protein histidine kinase, 0.0, 61% (CAD37055.1) 1266 aa	*Neurospora crassa *related to protein histidine kinase, 0.0, 61% (CAD37055.1) 1266 aa	Sln1p, 1e-56, 37% (NP_012119.1) 1220 aa	None	*Oryza sativa *putative histidine kinase, 4e-36, 27% (XP_463649.1) 1023 aa	*Neurospora crassa *related to protein histidine kinase, 0.0, 61% (CAD37055.1) 1266 aa	*Emericella nidulans *Two-component system protein B precursor NHK1, 0.0, 49% (Q9P4U6) 1070 aa	*Gibberella moniliformis *putative histidine kinase HHK5p, 0.0, 61% (AAR30125.1) 1150 aa	*Magnaporthe grisea *hypothetical protein MG07312.4 0.0, 57% (XP_367387.1) 1206 aa
Histidine kinase	tre39871	**VIII**	*Gibberella moniliformis *putative phytochrome-like histidine kinase PHY1p, 0.0, 65% (AAR30124.1) 1469 aa	*Gibberella moniliformis *putative phytochrome-like histidine kinase PHY1p, 0.0, 65% (AAR30124.1) 1469 aa	None	None	*Sorghum propinquum *phytochrome C, 1e-29, 25% (AAR33032.1) 1135 aa	*Neurospora crassa *phytochrome-1, 0.0, 62% (AAZ57422.1) 1536 aa	*Aspergillus nidulans *phytochrome, 0.0, 50% (CAI30283.1) 1280 aa	*Gibberella moniliformis *putative phytochrome-like histidine kinase PHY1p, 0.0, 65% (AAR30124.1) 1469 aa	*Magnaporthe grisea *hypothetical protein MG1342.4, 3e-34, 27% (XP_363416.1) 188 aa
Histidine kinase	tre44503	**IX**	*Gibberella moniliformis *putative histidine kinase HHK6p, 0.0, 67% (AAR30123.1) 1369 aa	*Gibberella moniliformis *putative histidine kinase HHK6p, 0.0, 67% (AAR30123.1) 1369 aa	None	hypothetical protein SPAC1834.08, 6e-50, 30% (CAB75776.1) 1639 aa	*Oryza sativa *putative sensor histidine kinase PK4, 4e-41, 40% (AAQ07255.1) 653 aa	*Neurospora crassa *hypothetical protein NCU2057.1, 0.0, 52% (XP_964013) 1319 aa	*Aspergillus fumigatus *sensor histidine kinase/response regulator, putative, 3e-45, 45% (EAL92328.1) 2333 aa	*Gibberella moniliformis *putative histidine kinase HHK6p, 0.0, 67% (AAR30123.1) 1369 aa	*Magnaporthe grisea *hypothetical protein MG01342.4, 0.0, 54% (EAA55691.1) 1388 aa
Histidine kinase	tre36042	**X**	*Gibberella moniliformis *putative histidine kinase HHK1p, 0.0, 71% (AAR30122.1) 2362 aa	*Gibberella moniliformis *putative histidine kinase HHK1p, 0.0, 71% (AAR30122.1) 2362 aa	None	None	*Oryza sativa *putative sensor kinase PK4, 3e-61, 36% (AAP07255.1) 653 aa	*Neurospora crassa *hypothetical protein, 0.0, 63% (XP_328262.1) 2476 aa	*Aspergillus fumigatus *sensor histidine kinase/response regulator, 0.0, 42% (EAL92328.1) 2333 aa	*Gibberella moniliformis *putative histidine kinase HHK1p, 0.0, 71% (AAR30122.1) 2362 aa	*Magnaporthe grisea *hypothetical protein MG06696.4, 0.0, 59% (XP_370199.1) 2580 aa
Histidine kinase	tre30271	**XI**	*Gibberella zeae *hypothetical protein FG00856.1, 0.0, 66% (EAA70449.1) 1521 aa	*Gibberella zeae *hypothetical protein FG00856.1, 0.0, 66% (EAA70449.1) 1521 aa	None	hypothetical protein SPAC1834.08, 3e-20 (CAB75776.1) 1639 aa	*Arabidopsis thaliana *cytokinin independent 2, 4e-17 (AAZ98829.1)	*Neurospora crassa *hypothetical protein NCU00939.1, 0.0, 53% (XP_325119.1) 1542 aa	*Aspergillus nidulans *histidine kinase G2, 7e-79, 32% (AAQ19474.1) 659 aa	*Gibberella zeae *hypothetical protein FG00856.1, 0.0, 66% (EAA70449.1) 1521 aa	*Magnaporthe grisea *hypothetical protein MG01227.4, 0.0, 51% (XP_363301.1) 1586 aa
Response regulator	tre124302	**RR**	*Gibberella zeae *hypothetical protein FG08948.1, 3e-100, 52% (EAA70828.1) 868 aa	*Gibberella zeae *hypothetical protein FG08948.1, 3e-100, 52% (EAA70828.1) 868 aa	Ssk1p, 4e-41, 43% (NP_013106.1) 712 aa	Mcs4, 3e-50, 58% (P87323) 522 aa	*Arabidopsis thaliana *cytokinin independent 2, 1e-11, 31% (AAZ98829.1) 922 aa	*Neurospora crassa *hypothetical protein, 4e-90, 45% (XP_329085.1) 1114 aa	*Aspergillus nidulans *hypothetical protein AN7697.2, 5e-67, 62% (XP_680966.1) 838 aa	*Gibberella zeae *hypothetical protein FG08948.1, 3e-100, 52% (EAA70828.1) 868 aa	*Magnaporthe grisea *hypothetical protein MG02897.4, 5e-78, 71% (XP_366821.1) 833 aa
Response regulator	tre44708	**RR**	*Gibberella zeae *hypothetical protein FG06359.1, 0.0, 61% (EAA73807.1) 587 aa	*Gibberella zeae *hypothetical protein FG06359.1, 0.0, 61% (EAA73807.1) 587 aa	Skn7p, 1e-21, 36% (P38889) 622 aa	Pombe response regulator Prr1, 2e-53, 30% (O14283) 539 aa	*Arabidopsis thaliana *heat shock factor 6, 4e-14, 39% (Q9SCW4) 299 aa	*Neurospora crassa *hypothetical protein, 3e-80, 42% (XP_331189.1) 555 aa	*Aspergillus nidulans *hypothetical protein AN3688.2, 4e-82, 39% (XP_661292.1) 475 aa	*Gibberella zeae *hypothetical protein FG06359.1, 0.0, 61% (EAA73807.1) 587 aa	*Magnaporthe grisea *hypothetical protein MG03516.4, 1e-83, 43% (XP_360973.1) 688 aa
Response regulator	tre31795	**RR**	*Gibberella zeae *putative response regulator receiver RIM15p, 0.0, 72% (AAR30133.1) 1916 aa	*Gibberella zeae *putative response regulator receiver RIM15p, 0.0, 72% (AAR30133.1) 1916 aa	Rim15p, 2e-110, 38% (NP_116620.1) 1770 aa	cek1, 5e-119, 43% (CAB40178.1) 1338 aa	*Arabidopsis thaliana *IRE homolog, 2e-47, 61% (BAB02708.1) 1398 aa	*Neurospora crassa *hypothetical protein 0.0, 62% (XP_327664.1) 1943 aa	*Aspergillus nidulans *hypothetical protein AN7572.2, 0.0, 54% (XP_680841.1) 2104 aa	*Gibberella zeae *putative response regulator receiver RIM15p, 0.0, 72% (AAR30133.1) 1916 aa	*Magnaporthe grisea *hypothetical protein MG00345.4, 0.0, 64% (XP_368899.1) 1952 aa
Histidine phosphotransferase	tre123344	**HPT**	*Gibberella zeae *putative histidine phosphotransferase HPT1p, 1e-44, 73% (AAR30118.1) 143 aa	*Gibberella zeae *putative histidine phosphotransferase HPT1p, 1e-44, 73% (AAR30118.1) 143 aa	Phosphorelay intermediate protein Ypd1p, 1e-10, 45% (NP_010046.1) 167 aa	Multistep phosphorelay regulator 1 MPR1, 2e-24, 47% (O94321) 295 aa	*Arabidopsis thaliana *Ahp1, 4e-5, 24% (BAA36335.1) 154 aa	*Neurospora crassa *hypothetical protein 1e-24, 52% (XP_327928.1) 145 aa	*Aspergillus fumigatus *histidine containing phosphotransmitter protein, 8e-31, 58% (EAL89760.1) 191 aa	*Gibberella zeae *putative histidine phospho-transferase HPT1p, 1e-44, 73% (AAR30118.1) 143 aa	*Magnaporthe grisea *hypothetical protein MG07173.4, 8e-31, 56% (XP_367248.1) 135 aa

Besides the histidine kinases as central components of the phosphorelay, also one histidine phosphotransferase (tre123344; IPR008207) and three response regulator receivers (tre124302, tre44708 and tre31795) were identified, two of which correspond to the *S. cerevisiae *RRs Ssk1p and Skn7p and could consequently have a function in osmosensing or response to oxidative stress, respectively (Table 4). Among them, tre124302 corresponds to the recently characterized *N. crassa *response regulator protein RRG-1 [87] and tre44708 represents the *H. jecorina *orthologue of RRG-2. tre31795 contains an N-terminal serine/threonine protein kinase domain additionally to the response regulator domain and is related to *S. cerevisiae *Rim15p. This kinase acts immediately downstream and under the control of the cAMP-dependent protein kinase. Overexpression of Rim15 partially induces a starvation response [114].

The presence of hybrid histidine kinases and multistep phosphorelays provides the fungus with a sophisticated means to integrate numerous environmental signals by differential regulation of the HKs and RRs. Since *H. jecorina*, as all other as yet analyzed fungi, only has one histidine phosphotransferase, this protein is likely to have a crucial regulatory function on several output pathways.

### Circadian rhythms and light response

Light is a crucial environmental factor for most living creatures. The presence or absence of light is connected to a plethora of fundamental influences on the life of an organism such as the intensity of harmful UV-light as well as changes in humidity and temperature. Anticipation of and appropriate adaptation to these changes inferred by sunrise or sunset can thus provide a significant evolutionary advantage (for reviews see [115,116]). In the case of fungi the presence of light also indicates growth over an exposed surface, where the encounter of a potential mating partner can be expected and successful dissemination or sexual development after disposal of spores and conidia is enabled. This might be one rationale behind the often reported effect of light on conidiation of fungi.

Photobiology in *H. jecorina *has only recently received closer attention because of the finding that cellulase gene expression is modulated by light [12]. Nevertheless, there is only limited information available on circadian rhythms and light response at the molecular level in this fungus. A summary on light induced development in *Trichoderma *species with an emphasis on sporulation has been provided by Betina and Farkas [117]. The main reported responses to illumination described so far are a rapid rise in the intracellular concentration of ATP, paralleled by an increase in the intracellular level of cyclic AMP [118-120], a burst of respiratory activity as indicated by oxygen uptake by dark-grown mycelia of *Trichoderma *immediately after the onset of illumination [121] and initiation of photoconidiation.

*H. jecorina *sporulates well both in light and darkness, albeit the amount of spores produced is lower in constant darkness. In *Trichoderma viride*, the production of conidiation rings depends on the frequency of irradiation and is not subject to circadian rhythm [117,122]. A similar phenomenon has been observed in *H. jecorina*: In the absence of light pulses, *H. jecorina *sporulates continuously without detectable formation of conidiation rings both in constant light or constant darkness (M. Schmoll, unpublished results).

The central components of the signaling pathways triggering circadian rhythms and light response have been studied extensively in *N. crassa *(for reviews see [123-125]). The most important input factors for the entrainment of the circadian clock are light and temperature, the blue light photoreceptor WC-1 (white collar-1) being essential for the circadian clock and other light responses and WC-2 (white collar-2) representing its partner in the light signaling pathway. Together these proteins form the white collar complex and both components have been shown to be required for all known light responses. This complex binds to the promotor of the clock gene *frequency *(*frq1*) and drives its expression. The respective gene product in turn is able to dampen expression of its own transcript by interaction with the white collar complex [124]. Besides these central components also the small LOV/PAS domain blue light sensor VIVID (VVD) plays important roles in light responses and photoentrainment of the clock [126,127]. The genome of *H. jecorina *comprises orthologues of *N. crassa *WC-1 (BLR1, AAV80185, E-value 0.0), WC-2 (BLR2, AAV80186, E-value 1e-118), VVD (ENV1, AY551804, E-value 1e-32) and FRQ (FRQ1, E-value 0.0). Thus the central components of the clock are available. The homologues of the *H. jecorina *photoreceptors BLR1 and BLR2 have been studied in the closely related *T. atroviride *and are essential for blue light induced conidiation [128,129], which is dependent on the carbon source [63]. However, it is important to note that in *T. atroviride *the *env1*-transcript could not be detected and the genomic sequence indicates that functional expression of this important light regulatory gene might be perturbed [13].

ENVOY (ENV1) represents the first signal transduction factor investigated in *H. jecorina *and was shown to be involved both in light response as well as in regulation of cellulase gene expression [12]. In addition, also dark-related functions as well as both positive and negative regulation of light-responsive genes by ENVOY has been reported [13]. Besides the PAS domain proteins described so far (see also section Two component phosphorelay systems), no additional PAS-domain proteins were identified.

Further *N. crassa *clock-associated proteins with orthologues found in *H. jecorina *include the E3-ubiquitin ligase FWD-1 (tre30183), which targets FRQ for degradation via the 26 S proteasome and the light inducible BLI-3 (tre42044). Also three potential class I photolyases were found, one of them representing the homologue of PHR1, which is rapidly (auto-) regulated by blue light in *Trichoderma harzianum *[130-132]. tre59726 shares considerable similarity with DASH-type cryptochromes from other organisms and is therefore likely to represent the *H. jecorina *homologue of the *Drosophila melanogaster *blue light dependent regulator of the circadian feedback loop CRY1 [133] (Table 5). Both PHR1 and tre59726 share high similarities with proteins in *N. crassa*, other fungi and numerous bacteria. In contrast, tre77473 shows E-values below 1e-95 to proteins in higher eukaryotes, especially the 6-4 photolyases of *Danio rerio *and *Xenopus laevis*, and uncharacterized proteins of several filamentous fungi, but seems to have no homologue in *N. crassa*.

**Table 5:** Putative photolyases and cryptochromes.

**Protein class**	**protein ID**	**Best hit overall**	***Drosophila***	**Animal**	**Plant (Viridiplantae limited)**	***Neurospora***	***Aspergillus***	***Fusarium***	***Magnaporthe grisea***	***Ustilago maydis***
DNA photolyase	tre77473	*Gibberella zeae *hypothetical proteinFG06765.1, 0.0, 71% (XP_386941.1)	RE11660p (phr6-4), 3e-95, 40% (AAL90322.1)	*Danio rerio *Cry5, 2e-102, 39% (AAH44204.1)	*Arabidopsis thaliana *UVR3 (UV repair defective 4), 2e-92, 36% (NP566520.1)	hypothetical protein NCU00582.1 (XP_965722.1), 3e-22, 23% (CRY homologue)	*Aspergillus clavatus *DNA photolyase, putative, 0.0, 58% (XP_001270768.1)	*Gibberella zeae *hypothetical proteinFG06765.1, 0.0, 71% (XP_386941.1)	hypothetical protein MGG_02071, 0.0, 57% (XP_365369.2)	hypothetical protein UM02144.1, 1e-135, 42% (XP_758291.1)
DNA photolyase	PHR1	*Hypocrea lixii *DNA photolyase 0.0, 83% (CAA08916.1)	RE11660p (phr6-4), 3e-35, 27% (AAL90322.1)	*Danio rerio *Cryptochrome DASH, 1e-40, 31% (AAH98514.1)	None	Deoxypyrimidine photolyase PHR-1, 0.0, 66% (P27526)	*Aspergillus clavatus *photolyase Phr1, putative, 0.0, 60% (XP_001269979.1)	*Fusarium oxysporum *photolyase 0.0, 75%, (AF500083.1)	hypothetical protein MGG_06836, 0.0, 66% (XP_370339.2)	hypothetical protein UM06079.1, 3e-111, 42% (XP_762226.1)
cryptochrome	tre59726	*Neurospora crassa *hypothetical protein NCU00582.1 (CRY homologue), 2e-116, 39% (Q7SI68)	photolyase, 3e-13, (BAA12067.1)	*Danio rerio *Cryptochrome DASH,1e-40, 26% (NP_991249.1)	*Arabidopsis thaliana *CRY3 (Cryptochrome 3), 2e-25, 26% (NP_568461.2)	hypothetical protein NCU00582.1, 2e-116, 39% (Q7SI68)	*Aspergillus oryzae *unnamed protein product, 2e-13, 37% (BAE61131.1)	*Gibberella zeae *putative cryptochrome DASH, 3e-100, 36% (Q4I1Q6)	hypothetical protein MGCH7_ch7g783, 9e-112, 38% (XP_001522685.1)	hypothetical protein UM01131.1, 4e-42, 28% (XP_757278.1)

Nearest neighbours of the respective organisms are listed along with E-value as determined by NCBI blastp search, percent identities within fragment and GenBank accession number.

Besides the casein kinase I HHP1, the *N. crassa *homologue of which is a homologue of *D. melanogaster *DOUBLETIME and involved in phosphorylation of FRQ [134], two further candidate casein kinase I proteins have been detected (tre30360 and tre12219). One of them (tre12219) shows highest similarity to a vertebrate casein kinase and seems to have no corresponding homologue in *N. crassa*, since the best hit again was HHP1 (Table 6). This protein is also highly similar to *D. melanogaster *DOUBLETIME and could thus indicate an expansion of this group in *H. jecorina*. In contrast to *N. crassa *for which a crucial role of casein kinase II in phosphorylation of FRQ has been reported, *H. jecorina *not only possesses two casein kinase II beta (regulatory) subunits (CKB1 and CKB2) but also two casein kinase II alpha (catalytic) subunits (KC2A and tre38035). tre38035 has in most cases the same homologues as KC2A, but the best hit is a casein kinase II of *Coccidioides imitis*. Hence also in this group of casein kinases an expansion may have taken place. A further putative kinase involved in phosphorylation of FRQ, the *N. crassa *calcium/calmodulin protein kinase CAMK has two orthologues in *H. jecorina*: tre44095 and tre18772. Consequently, these apparent expansions in proteins involved in phosphorylation and thus regulation of FRQ suggest that a more sophisticated regulation of the regulatory cycles in which this protein participates may occur. Characterization of FRQ in *H. jecorina *will elucidate whether this presumably more complex regulation is reflected in a broader spectrum of output pathways. Moreover a glycogen synthase kinase 3 homologue (GSK3) related to *D. melanogaster *SHAGGY, which is involved in phosphorylation of the central oscillator component TIM [133] was detected. Also a putative homologue of TIM (TIMELESS) is available in *H. jecorina *(tre53569).

**Table 6:** Casein kinases.

**Protein class**	**protein ID**	**Best hit overall**	***Drosophila***	**Animal**	**Plant (Viridiplantae limited)**	***Neurospora***	***Aspergillus***	***Fusarium***	***Magnaporthe grisea***	***Ustilago maydis***
casein kinase I	HHP1	*Gibberella zeae *conserved hypothetical protein, 0.0, 96% (XP_388907.1)	*Drosophila melanogaster *double-time 2e-132, 69% (AAF27857.1)	*Rattus norvegicus *casein kinase 1 epsilon-2, 2e-144, 65% (BAB32922.1)	*Oryza sativa *hypothetical protein OsJ_008513 4e-141, 60% (EAZ25030.1)	*Neurospora crassa *casein kinase I homolog HHP1, 4e-173, 82% (XP_965825.1)	*Aspergillus terreus *casein kinase I isoform epsilon, 0.0, 88% (XP_001209612.1)	*Gibberella zeae *conserved hypothetical protein, 0.0, 96% (XP_388907.1)	casein kinase I, 0.0, 88% (XP_366753.1)	Hypothetical protein UM00584.1, 3e-161, 88% (XP_756731.1)
casein kinase I	tre30360	*Gibberella zeae *conserved hypothetical protein, 0.0, 91% (XP_390242.1)	*Drosophila melanogaster *gilgamesh CG6963-PA, 6e-105, 61% (NP732123.1)	*Mus musculus *casein kinase 1, gamma 3, 3e-108, 65% (NP_690022.2)	*Oryza sativa *casein kinase I like, 2e-95, 45% (BAB92346.1)	*Neurospora crassa *probable casein kinase I cki2, 5e-177, 72% (XP_957620.1)	*Aspergillus niger *hypothetical protein An18g06050, 0.0, 76% (XP_001399056.1)	*Gibberella zeae *conserved hypothetical protein, 0.0, 91% (XP_390242.1)	conserved hypothetical protein, 0.0, 81% (XP_362514.2)	Hypothetical protein UM00274.1, 1e-154, 86% (XP_756421.1)
casein kinase I	tre12219	*Bos taurus *casein kinase 1, epsilon, 4e-95, 56% (NP_001071577.1)	*Drosophila melanogaster *double-time 8e-87, 52% (AAF27857.1)	*Danio rerio *casein kinase 1, delta, 1e-94, 56% (NP_955877.1)	*Beta vulgaris *casein kinase, 1e-90, 50% (ABM55249.1)	*Neurospora crassa *casein kinase I homolog HHP1, 1e-89, 52% (XP_965825.1)	*Aspergillus fumigatus *casein kinase I, putative, 4e-94, 51% (XP_749388.1)	*Gibberella zeae *conserved hypothetical protein, 1e-94, 55% (XP_388907.1)	hypothetical protein MGCH7_ch7g585, 3e-94, 55% (XP_001522482.1)	hypothetical protein UM0584.1, 6e-92, 55% (XP_756731.1)
casein kinase II	KC2A	*Gibberella zeae *Casein kinase II, alpha chain, 3e-173, 95% (XP_380853.1)	*Drosophila melanogaster *Casein kinase II subunit alpha, 7e-122, 70%, (P08181)	*Danio rerio *similar to alpha subunit of casein kinase II, 2e-131, 68% (XP_699829.1)	*Arabidopsis thaliana *casein kinase II catalytic subunit, 1e-140, 73% (BAA01091.1)	*Neurospora crassa *casein kinase II subunit alpha, 2e-165, 91%, (Q8TG13)	*Aspergillus niger *hypothetical protein An16g08010, 1e-158, 90% (XP_001398099)	*Gibberella zeae *Casein kinase II, alpha chain, 3e-173, 95% (XP_380853.1)	casein kinase II, 2e-168, 93%, (XP361153.1)	hypothetical protein UM01180.1, 3e-141, 78% (XP_757327.1)
casein kinase II	tre38035	*Coccidioides imitis *casein kinase II, alpha chain, 1e-78, 52% (XP_001248389.1)	*Drosophila melanogaster *Casein kinase II subunit alpha, 1e-66, 47%, (P08181)	*Danio rerio *similar to alpha subunit of casein kinase II, 2e-77, 48% (XP_699829.1)	*Arabidopsis thaliana *casein kinase II catalytic subunit, 1e-73, 48% (BAA01091.1)	*Neurospora crassa *casein kinase II subunit alpha, 2e-76, 51%, (Q8TG13)	*Aspergillus nidulans *casein kinase II subunit alpha, 1e-76, 51% (XP_659089.1)	*Gibberella zeae *casein kinase II, alpha chain 4e-74, 50% (XP_380853.1)	casein kinase II, 2e-77, 52%, (XP361153.1)	hypothetical protein UM01180.1, 2e-71, 48% (XP_757327.1)
casein kinase II	CKB1	*Gibberella zeae *hypothetical protein FG08607.1, 5e-146, 80% (XP_388783.1)	*Drosophila melanogaster *casein kinase II beta subunit, 1e-45, 45% (NP_511131.2)	*Homo sapiens *casein kinase 2, beta polypeptide, 2e-47, 44% (CAI183941.1)	*Arabidopsis thaliana *CKB4 (Casein kinase II beta subunit 4), 5e-38, 42% (NP_850421.1)	*Neurospora crassa *casein kinase II subunit beta-1, 9e.136, 75% (Q8TG12)	*Aspergillus terreus *casein kinase II beta 1 subunit, 1e-97, 56% (XP_001214781.1)	*Gibberella zeae *hypothetical protein FG08607.1, 5e-146, 80% (XP_388783.1)	hypothetical protein MGG_00446, 3e-121, 71% (XP_368798.1)	hypothetical protein UM01094.1, 1e-61, 46% (XP_757241.1)
casein kinase II	CKB2	*Gibberella zeae *hypothetical protein FG01288.1, 3e-126, 91% (XP_381464.1)	*Drosophila melanoaster *casein kinase II beta subunit, 1e-56, 48% (NP_511131.2)	*Canis familiaris *similar to casein kinase 2, beta subunit, 9e-59, 50% (XP_532075.2)	*Zea mays *protein kinase CK2, regulatory subunit CK2B1, 2e-58, 53% (AAG36869.1)	*Neurospora crassa *casein kinase II subunit beta-2, 3e-110, 87% (Q8TG11)	*Aspergillus terreus *casein kinase II beta 2 subunit, 9e-96, 78% (XP_001210278.1)	*Gibberella zeae *hypothetical protein FG01288.1, 3e-126, 91% (XP_381464.1)	hypothetical protein MGG_05651, 3e-72, 81% (XP_360277.2)	hypothetical protein UM06107.1, 4e-68, 58% (XP_762254.1)
casein kinase II	GSK3	*Gibberella zeae *hypothetical protein FG07329.1, 0.0, 92% (XP_387505.1)	*Drosophila melanogaster *shaggy, 2e-129, 64% (NP_001036259.1)	*Homo sapiens *glycogen synthase kinase-3 beta, 3e-133, 63% (P49841)	*Triticum aestivum *shaggy-like kinase, 7e-122, 63% (BAF36565.1)	*Neurospora crassa *glycogen synthase kinase-3, 0.0, 89% (AAS68519.1)	*Aspergillus clavatus *glycogen synthase kinase (Skp1), 0.0, 83% (XP_001270035.1)	*Gibberella zeae *hypothetical protein FG07329.1, 0.0, 92% (XP_387505.1)	glycogen synthase kinase, 0.0, 90% (ABA02071.1)	*Ustilago hordei *glycogen synthase kinase, 3e-149, 66% (CAJ42002.1)

Nearest neighbours of the respective organisms are listed along with E-value as determined by NCBI blastp search, percent identities within fragment and GenBank accession number.

Interestingly, although no responses to red light have been reported for *H. jecorina *so far, its genome comprises a reading frame related to *A. nidulans *VELVET and one candidate phytochrome like histidine kinase (tre39871). Despite the fact that VELVET has initially been reported for its involvement in red-light initiated conidiation in *A. nidulans *[135,136], recently functions exceeding developmental regulation, namely repression of penicillin biosynthesis in this fungus [137] have been shown. Given the lack of known red light responses, functions beyond regulation of development are also conceivable for tre39871.

It is intriguing that despite the presence of homologues to the major determinants of circadian rhythmicity known from other organisms, no such rhythmicity is obvious in *H. jecorina*, at least with respect to the formation of conidial rings in the absence of light pulses. Nevertheless, devoid of molecular data on potential rhythmic expression of at least a few genes, it is still possible that a circadian rhythm is operative, but that the output of the related signaling pathway is disconnected from conidiation. In this respect it is also interesting that *H. jecorina *only has one of the three best studied output genes of circadian rhythms described in *N. crassa *[138,139]: While the geranylgeranyl pyrophosphate synthetase *al-3*, which is involved in carotenoid biosynthesis, has a highly similar homologue in *H. jecorina *(tre14246), no homologues to *al-1 *and *al-2 *have been detected, which is a further hint as to the presence of different targets of the output pathways of circadian rhythmicity in *N. crassa *and *H. jecorina*. On the other hand, both for *H. jecorina *as well as for the closely related *H. atroviridis *it has been shown that numerous genes are regulated by light, which thus could be clock components [13,140].

### Calcium signaling

Calcium plays an important role in intracellular signaling processes in eukaryotic cells [141,142]. The respective signal transduction machinery comprises Ca^2+^-permeable channels, Ca-pumps and -transporters as well as numerous other Ca^2+ ^signaling proteins. These components cooperate to transduce various external signals [143,144]. The interaction of calcium signaling with the cAMP-pathway via regulation of adenylyl cyclase [145] suggests a rather widespread function of calcium in regulation of cellular events. In this signaling cascade the calcium binding protein calmodulin represents a central component which in its Ca^2+ ^-activated form is able to activate or inactivate a number of enzymes including protein kinases and phosphoprotein phosphatases and is important for regulation of stress responses in fungi [146]. A comprehensive overview of calcium signaling proteins is available for *N. crassa*, *M. grisea *and *S. cerevisiae *[146]. For *H. jecorina *it was shown that calmodulin (CAM1) is required for formation and secretion of xylanases [14]. Further downstream components found in the genome of *H. jecorina *include a homologue of the Ca^2+^/calmodulin dependent Ser/Thr phosphatase calcineurin, which consists of a catalytic subunit, calcineurin A (CNA1) and the regulatory subunit, calcineurin B (CNAB). In *N. crassa*, the function of calcineurin was determined to be the regulation of hyphal tip growth and branching [147]. Also a homologue of the *N. crassa *Ca^2+^/calmodulin-dependent protein kinase CAMK-1 (tre44095), which plays a role in regulation of the circadian clock in this fungus by phosphorylation of FRQ [148] was found. 7 further putative Ca^2+ ^or calmodulin binding proteins have been detected in the genome of *H. jecorina *(Table 7).

**Table 7:** Proteins involved in Calcium signaling

**class of protein**	**ID**	**subtype**
**Phospholipase C**	tre60194 tre75311 tre37143 tre61746 PLC-E	

**Calmodulin**	CAM1 tre65659	

	CNA1	catalytic subunit
**Calcineurin**		
	CNAB	regulatory subunit

**Ca2+ and/or CaM binding proteins**	CMK2 tre18772 tre44095 tre119679 tre44531 tre64196 tre42143 tre33128	

**Calnexin/Calreticulin**	tre43732	

**Calpactin I heavy chain**	tre21646 tre29619	

Calcium signaling is mediated via controlled release of Ca^2+ ^from internal stores. The first regulatory component in the respective cascade is phospholipase C (PLC), which becomes activated by extracellular receptors and catalyzes the hydrolysis of phosphatidylinositol 4,5-bisphosphate to diacylglycerol and inositol 1,4,5-trisphosphate. Diacylglycerol represents the physiological activator of protein kinase C and inositol 1,4,5-trisphosphate induces the release of Ca^2+ ^from internal stores [149]. Thus phospholipase C has a central function in a transmembrane signal transduction mechanism which is known to regulate several cellular processes such as metabolism, secretion and growth. *H. jecorina *has four phospholipase C homologues showing the characteristics of eukaryotic phospholipase C proteins, but also one (PLC-E, ABG20593) with similarities to predominantly prokaryotic phospholipase C proteins. The latter one seems to have no orthologue in *Neurospora crassa *(Tables 8 and 9). However, no such protein has yet been characterized from fungi and since bacterial PLCs are reported to have different enzymatic characteristics than eukaryotic PLCs [150], their possible involvement in signal transduction remains to be elucidated. Bacterial PLCs do not require Ca^2+ ^for their activity and are considerably smaller than their eukaryotic counterparts. Such PLCs have been shown to play a role in virulence in pathogenic bacteria, but in many cases their precise function remains elusive. Interestingly, it was suggested that bacterial PLCs might be descendants from eukaryotic PLCs that were incorporated by the bacteria during evolution and that the loss of requirement for a Ca^2+ ^cofactor might have been evolutionary beneficial for bacteria living as intracellular parasites [150].

**Table 8:** Ca^2+ ^permeable channels, Cation pumps and Ca^2+ ^transporters

**class of protein**	**ID**	**subtype**
**Ca2+ permeable channel**	tre23028 tre37060 tre74057	

**Cation pumps**	tre43358 tre75347 tre58952 tre62362 tre81536 tre122972 tre81430 tre23221 tre41593 tre123183	

	tre79599 tre55595	Ca2+/proton exchangers
	
**Ca2+ transporters**	tre19432 tre56744 tre82544 tre68169 tre62835 tre76557 tre4171	Ca2+/Na+ exchangers

**Table 9:** Phospholipase C proteins.

**protein ID**	**Best hit overall**	***Neurospora***	**Prokaryote**	***Fusarium***	**Animal**
tre60194	*Gibberella zeae *hypothetical protein FG05898.1, 0.0, 56% (XP_386074.1)	*Neurospora crassa *probable phosphoinositide-specific phospholipase C NCU01266.1, 0.0, 43% (XP_960744.1)	None	*Gibberella zeae *hypothetical protein FG05898.1, 0.0, 56% (XP_386074.1)	*Macaca mulatta *similar to phospholipase C, 4e-73, 29% (XP_001091488.1)
tre75311	*Phaeosphaeria nodorum *hypothetical protein SNOG_09944, 0.0, 58% (EAT82279.1)	*Neurospora crassa *hypothetical protein NCU09655.1, 4e-82, 48% (EAA29485.1)	None	*Gibberella zeae *hypothetical protein FG05060.1, 0.0, 56% (XP_385236.1)	*Rattus norvegicus *phospholipase C, delta 1, 2e-41, 28% (P10688)
tre37143	*Gibberella zeae *hypothetical protein FG8790.1, 3e-164, 50% (XP_388966.1)	*Neurospora crassa *phospholipase C NCU06245.1, 1e-98, 39% (AAZ23804.1)	None	*Gibberella zeae *hypothetical protein FG8790.1, 3e-164, 50% (XP_388966.1)	*Homo sapiens *PLCD3 protein, 7e-35, 27% (AAH10668.2)
tre61746	*Gibberella zeae *hypothetical protein FG06693.1, 0.0, 52% (XP_386869.1)	*Neurospora crassa *phospholipase C NCU06245.1, 9e-160, 46% (AAZ23804.1)	None	*Gibberella zeae *hypothetical protein FG06693.1, 0.0, 52% (XP_386869.1)	*Rattus norvegicus *similar to phospholipase C, delta 3, 1e-42, 32% (XP_221004.4)
PLC-E	*Gibberella zeae *hypothetical protein FG11236.1, 0.0, 62% (XP_391412.1)	None	*Burkholderia dolosa *Phospholipase C, 1e-68, 33% (EAY67741.1)	*Gibberella zeae *hypothetical protein FG11236.1, 0.0, 62% (XP_391412.1)	None

Nearest neighbours of the respective organisms are listed along with E-value as determined by NCBI blastp search, percent identities within fragment and GenBank accession number.

For signal dependent alteration of Ca^2+ ^concentration *H. jecorina *has both calcium/sodium as well as calcium/proton antiporters, and calcium permeable channels available (Table 8). As for further components producing second messengers it is interesting to note that in contrast to *N. crassa *[25], *H. jecorina *indeed possesses a putative sphingosine kinase (tre68412) which synthesizes sphingosine-1-phosphate.

### Ras-like GTPases

The superfamily of small Ras-like GTPases comprises several subgroups (a subgroup hierarchy is given on NCBI CDD for domain cd00876 (Ras): [151]) and regulate various cellular signaling pathways including cell growth, differentiation, proliferation and apoptosis [152]. These proteins represent monomeric GTPases that function as molecular switches by cycling between an inactive, GDP-bound and an active GTP-bound state. Activation of Ras proteins is stimulated by guanine nucleotide exchange factors (GEFs), which promote exchange of GDP for GTP hence inducing a conformational change that permits interaction with downstream effectors. The duration of the active phase of Ras is limited by its intrinsic GTPase activity, which is strongly accelerated by GTPase activating proteins (GAPs). Thus GAPs can rapidly terminate the respective transduced signal [153,154].

Numerous studies dealt with the elucidation of the function of Ras-like GTPases in *S. cerevisiae*. In this organism, the Ras proteins are presumed to mediate signaling to adenylate cyclase [155,156], although this function has been debated thereafter [66] and the same is valid for a potential activation of Ras2 by the G-protein coupled receptor Gpa2 in yeast. Nevertheless a connection of Ras proteins to the cAMP-pathway is obvious and a role for Cdc25 and Ras in signaling of glucose availability has been suggested ([66] and references therein). However, for *A. nidulans *RasA and cAMP signaling are reported to proceed independently during germination [157]. A further signaling system targeted by Ras-GTPases is that of the MAP kinases. Here especially for the pheromone pathway an involvement of Cdc42, which activates Ste20 in yeast has been shown [71]. Ste20, which has an orthologue in *H. jecorina *(tre104364), thereby triggers activation of the pheromone response-MAP kinase cascade. Moreover the two component signaling response regulator Skn7 and the protein kinase Pkc1 (putative *H. jecorina *orthologues tre44708 and PKC1, respectively) are downstream effectors of Rho1-GTP [158]. Hence all three MAPkinase pathways postulated for *H. jecorina *could be subject to regulation by Ras-GTPases. Although detailed studies of Ras-like GTPases in filamentous fungi are scarce, potential indications as to the functions of at least some of these proteins can be deduced from their interaction characteristics and roles in yeast.

The putative homologues to the members of the Ras superfamily of small GTPases as well as GEFs and GAPs found in the genome of *H. jecorina *were grouped into the respective subgroups according to the presence of the characteristic domains as determined by InterPro [159] and NCBI CDD-search [160] as well as according to their *S. cerevisiae *orthologues and their targets (in case of GEFs and GAPs) (Table 10).

**Table 10:** The superfamily of Ras small GTPases

**Ras subfamily**	**Ras GTPase**	**RasGEF**	**RasGAP**
	YPT1		tre22132
			
	tre17707 tre30910		tre121349
			
	tre44333	tre75397	
	
**Rab**	tre44010		tre65982 tre44704 tre44251
	
	tre33269 tre31007 tre42061 tre42834 tre29515		
	
			tre35375

	tre21294 tre106302 tre41009 tre5278		
	
**Rho**	CDC42	tre30184	tre74848
	
	GEM1		
	
	tre53562 RHO3		tre14881 tre46043
	
	RHO1	tre37105	tre80713

	tre107035 tre66480		
	
	RAS1		
		tre34726	tre61408 tre22757
**Ras**	RAS2		
		tre107369	
	
	RSR1 (Rap-like)		tre81785
	
		tre67275	
			
		tre70548	

**Ran**	RAN1		

	tre28367 tre43177		
		
	tre29890	tre28378	
		
**Arf-like**	tre45468	tre29839	
		
	tre38187	tre37547	
		
	tre42316 SAR1		

**Rac**	RAC1		

**undetermined**			tre62426 tre37068

The respective small GTPases are grouped according to their assigned subfamily as suggested by the presence of appropriate protein domains determined by InterPro and NCBI CDD search as well as due to their similarity to known orthologues in GenBank. Guanine nucleotide exchange factors (GEFs) and GTPase activating proteins (GAPs) have been associated with the respective GTPase(s), provided that the regulatory target of the orthologue in yeast was known.

Interestingly, *H. jecorina *has a considerable number of these small GTPases at its disposal, although due to the lack of comprehensive studies in other fungi, this does not necessarily indicate an expansion of this group. In Table 10 an overview of the groups present is given along with the putative modulators (GEFs and GAPs), if a correlation based on the yeast orthologues (GenBank, NCBI) was possible. Consequently *H. jecorina *has at least 10 Rab-type GTPases putatively modulated by 1 GEF and 6 GAPs, 9 Rho-type GTPases targeted by 2 GEFs and 4 GAPs, 5 Ras-type GTPases subject to (de)activation by 4 GEFs and 3 GAPs, 7 Arf (ADP-ribosylation factor like) GTPases possibly influenced by 3 GEFs as well as one Ran-type and one Rac-type GTPase, for which no regulatory factors have been identified. Furthermore two GAPs have been found that could not be assigned to a specific group.

In particular the orthologues of the Ras-subtype GTPases RAS1 and RAS2 together with their modulators (tre34726, tre107369, tre61408, tre22757) are reported to influence the cAMP dependent protein kinase A [66] in *S. cerevisiae *and are involved in a G-protein/cAMP/protein kinase A signaling pathway regulating aflR expression in *A. nidulans *[161]. They could therefore have a function in the processes connected to such a pathway in *H. jecorina *such as cellulase gene expression or light response. Moreover a function in regulation of conidial germination, morphology and asexual development has been shown in *A. nidulans *[157,162-164]. Also two Rho-type GTPase orthologues of *H. jecorina*, RHO1 and CDC42, which have functions upstream of MAP kinase pathways in *S. cerevisiae *[71] might also in *H. jecorina *establish an important regulatory bridge between cell surface receptors and this crucial signaling system. Given the high number of Ras-superfamily genes found in the *H. jecorina *genome and the numerous putative functions with predicted links to almost every signaling system studied so far, elucidation of their roles in signal transduction could contribute considerably to a better understanding of some as yet enigmatic phenomena.

## Conclusion

Being an industrial workhorse, interest in signal transduction processes of *H. jecorina *has been limited so far. However, given the intriguing uniqueness of the enzymatic machinery of this fungus [7], it is tempting to speculate that sophisticated and efficient regulation of the surprisingly small number of degradative enzymes has enabled *H. jecorina *to efficiently compete in nature. Therefore an equally efficient system for perception and interpretation of environmental signals is indispensable. Although signal transduction pathways of *H. jecorina *are largely comparable with those of other filamentous fungi, also in several cases expansions of certain classes and missing of others has been detected. These alterations as well as the global signaling network optimized for survival in nature despite certain enzymatic shortcommings is likely to provide insights into crucial environmental cues causing regulatory adjustments in *H. jecorina*, which can be exploited to improve industrial fermentations.

## Methods

### Genome assembly information

The *Hypocrea jecorina *QM6a DNA sequence and protein predictions of the *Trichoderma reesei *genome database ver2.0 by the Joint Genome Institute  were used for this study (AAIL00000000). For comparison also the genome databases at the Joint Genome institute server [165] of the Fungal Genome Initiative [166], especially those of *Aspergillus nidulans *[167], *Neurospora crassa *[168], and *Magnaporthe grisea *[169] were used.

### Gene identification

In order to identify a specific gene, the putative orthologue of *N. crassa *[25] was used for a TBLASTN search on the *T. reesei *genome database. If no orthologue was available in *N. crassa*, the orthologue of the organism in which the respective protein had been characterized was used. In cases where the assignment to a certain orthologue was unclear (especially if the similarity was poor), the predicted protein model of *T. reesei *was again used for a TBLASTN search in the respective genome to check whether the respective protein was the true orthologue of the protein used for the identification. In some cases the model as provided by the automatic annotation was adjusted manually, especially if the start- or stop codon was missing or the protein model differed significantly from orthologues in other fungi. The sequence information, protein- and domain predictions were used as provided on the genome database homepage and crosschecked by NCBI CDD search [170], InterProScan [159] and NCBI Blastp [171,172].

Genes and encoded proteins were denominated according to the guidelines for *T. reesei *(*H. jecorina*) genome annotation: the name contains the prefix "tre" followed by the protein ID of the best model as suggested by *in silico *modelling and annotation within the Genome Browser of the *Trichoderma reesei *genome database ver2.0 or as determined manually by the respective annotator. If amino acid similarity of the respective model to a characterized protein exceeds 80%, these proteins are considered homologues and the *H. jecorina *protein is named after the homologous protein in three letter code followed by a number. In any case, *N. crassa *denomination of homologous proteins is preferred. The *Trichoderma reesei *genome database ver2.0 is publicly accessible free of charge.

### Sequence alignment and phylogenetic analysis

Sequence alignments were performed using ClustalX (1.81) [173] and the alignment was manually adjusted by the aid of Genedoc. The evolutionary relationships were inferred using the Minimum Evolution method [174] and the software MEGA4. The percentage of replicate trees in which the associated proteins clustered together in the bootstrap test (500 replicates) are shown next to the branches [175]. The trees are drawn to scale, with branch lengths in the same units as those of the evolutionary distances used to infer the phylogenetic tree. The evolutionary distances were computed using the Poisson correction method [176] and are in the units of the number of amino acid substitutions per site. The ME tree was searched using the Close-Neighbor-Interchange (CNI) algorithm [177] at a search level of 1. The Neighbor-joining algorithm [178] was used to generate the initial tree. All positions containing gaps and missing data were eliminated from the dataset (Complete deletion option). There were a total of 128 positions in the final dataset. Phylogenetic analyses were conducted in MEGA4 [179].
